# Common Variants in Left/Right Asymmetry Genes and Pathways Are Associated with Relative Hand Skill

**DOI:** 10.1371/journal.pgen.1003751

**Published:** 2013-09-12

**Authors:** William M. Brandler, Andrew P. Morris, David M. Evans, Thomas S. Scerri, John P. Kemp, Nicholas J. Timpson, Beate St Pourcain, George Davey Smith, Susan M. Ring, John Stein, Anthony P. Monaco, Joel B. Talcott, Simon E. Fisher, Caleb Webber, Silvia Paracchini

**Affiliations:** 1 Wellcome Trust Centre for Human Genetics, University of Oxford, Oxford, United Kingdom; 2 MRC Functional Genomics Unit, Department of Physiology, Anatomy & Genetics, University of Oxford, Oxford, United Kingdom; 3 MRC Centre for Causal Analyses in Translational Epidemiology, University of Bristol, Bristol, United Kingdom; 4 School of Social and Community Medicine, University of Bristol, Bristol, United Kingdom; 5 The Walter and Eliza Hall Research Institute of Medical Research, Melbourne, Australia; 6 Department of Physiology, University of Oxford, Oxford, United Kingdom; 7 School of Life and Health Sciences, Aston University, Birmingham, United Kingdom; 8 Max Planck Institute for Psycholinguistics, Nijmegen, The Netherlands; 9 Donders Institute for Brain, Cognition and Behaviour, Radboud University, Nijmegen, The Netherlands; 10 School of Medicine, University of St Andrews, St Andrews, United Kingdom; University of California Los Angeles, United States of America

## Abstract

Humans display structural and functional asymmetries in brain organization, strikingly with respect to language and handedness. The molecular basis of these asymmetries is unknown. We report a genome-wide association study meta-analysis for a quantitative measure of relative hand skill in individuals with dyslexia [reading disability (RD)] (*n* = 728). The most strongly associated variant, rs7182874 (*P* = 8.68×10^−9^), is located in *PCSK6*, further supporting an association we previously reported. We also confirmed the specificity of this association in individuals with RD; the same locus was not associated with relative hand skill in a general population cohort (*n* = 2,666). As PCSK6 is known to regulate NODAL in the development of left/right (LR) asymmetry in mice, we developed a novel approach to GWAS pathway analysis, using gene-set enrichment to test for an over-representation of highly associated variants within the orthologs of genes whose disruption in mice yields LR asymmetry phenotypes. Four out of 15 LR asymmetry phenotypes showed an over-representation (FDR≤5%). We replicated three of these phenotypes; situs inversus, heterotaxia, and double outlet right ventricle, in the general population cohort (FDR≤5%). Our findings lead us to propose that handedness is a polygenic trait controlled in part by the molecular mechanisms that establish LR body asymmetry early in development.

## Author Summary

Humans have developed a population level bias towards right-handedness for tool-use. Understanding the genetic basis of handedness can help explain why this bias exists and may offer clues into the evolution of handedness and brain asymmetry. We have tested for correlation between relative hand skill and hundreds of thousands of genetic variants in a cohort of individuals with reading disability. The strongest associated variant is in the gene *PCSK6*, an enzyme that cleaves NODAL into an active form. NODAL plays a key role during the establishment of left/right (LR) asymmetry in diverse species, from snails to mammals. *Pcsk6* knock-out mice display LR asymmetry defects like heterotaxia (abnormal organ positioning). We uncovered further variants associated with relative hand skill in the human versions of genes that also cause the LR asymmetry phenotypes heterotaxia, and situs inversus (reversal of organ asymmetry) when knocked out in mice. These results replicate in an independent general population cohort without reading disability. We propose that handedness is under the control of many variants, some of which are in genes that also contribute to the determination of body LR asymmetry.

## Introduction

Humans display such striking cerebral asymmetries that we have been labeled, “The Lopsided Ape” [1]. The prevalence of right-handedness for tool use in excess of 85% across all human societies implies left-hemisphere dominance for controlling hand preference [2], [3]. The same degree of collective population bias is not observed in the other great apes [4], [5], suggesting right-handedness evolved along the human lineage [6]. Studies of fossil hominins suggest that the evolution of right-handedness dates back over half a million years [7], and is shared with *Homo neanderthalensis* [8], [9] and *Homo heidelbergensis* [10].

Handedness correlates with asymmetries of both the motor cortex and language-related areas of the brain, such as the planum temporale [11]–[13]. Furthermore, there is a weak correlation between language lateralization and handedness: in a study of 326 individuals, 96% of strong right-handers, as compared with 73% of strong left-handers, show left-hemisphere dominance for language [14]. Because language, like handedness, evolved primarily along the human lineage, it has been proposed that the two evolved together [15]. It has also been hypothesized that atypical cerebral asymmetries are associated with neurodevelopmental disorders. Although many associations appear to be spurious [16], dyslexia has been associated with structural and functional asymmetry differences, particularly in language related regions like the superior temporal gyrus (which includes the planum temporale) [17], [18]. Schizophrenia has been associated with both reduced planum temporale asymmetry [19]–[21] and an increased prevalence of left-handedness [22].

Both cerebral asymmetry and handedness are heritable traits, with additive genetic effects estimated to account for about a half and a quarter of the variance, respectively [23]–[25]. Heritability estimates are consistent with theories that propose a single causal gene underlies handedness [26]–[28], however population genomics theory predicts that a significant proportion of the genetic variance in common traits is the results of multiple common variants of small effect [29]–[31]. Indeed, the first direct molecular studies of handedness support the view that it has a complex multifactorial basis. To date, positional cloning and candidate gene studies have implicated *LRRTM1* and *AR*, each of which would explain only a small proportion of the genetic variance in handedness. The imprinted *LRRTM1* at 2p12 has been associated with both relative hand skill and schizophrenia [32]. A candidate gene approach found that CAG repeat length in the X-linked androgen receptor (*AR*) has opposite effects on the probability of left-handedness in males and females [33]. The same polymorphism in *AR* was found to be associated with ‘mixed-handedness’ in an independent cohort [34]. A genome-wide association study (GWAS) of 4,268 individuals for hand preference [35] found no single nucleotide polymorphisms (SNPs) associated at *P*≤5×10^−8^, the standard genome-wide threshold for significance. Based on GWAS and linkage studies, both published and unpublished, it has been estimated that that at least 40 loci underlie the genetic basis of handedness [36].

We recently identified the first association with handedness through a GWAS for relative hand skill (peg-board task, see **Materials and Methods**) [37]. The associated variant, rs11855415, is located in an intron of the proprotein convertase subtilisin/kexin type 6 (*PCSK6*, also known as *PACE4*). PCSK6 is a protease that cleaves NODAL proprotein into an active form. NODAL determines the development of left/right (LR) asymmetry in bilaterians from snails to vertebrates [38], [39]. Mice in which *Pcsk6* is disrupted display asymmetry defects like heterotaxia (abnormal distribution of visceral organs) [40].

In our previous study, we analyzed a cohort of individuals originally selected for reading disability (RD; dyslexia) that were characterized with handedness measures. The most highly associated SNP, rs11855415, was then genotyped in two independent cohorts with RD, reaching genome-wide significance in the combined meta-analysis [37]. However, the association was specific to individuals with RD; rs11855415 was not associated with hand skill in a general population cohort unaffected with RD. A study of an independent general population cohort has identified a variable number tandem repeat (VNTR), close to rs11855415, associated with degree of handedness, providing further evidence that *PCSK6* is important in the development of handedness [41].

Here we have genotyped the replication cohorts from our original study genome-wide, and perform a meta-analysis after imputation with reference panels from the 1000 Genomes Project [42]. The *PCSK6* locus is the only genome-wide significant signal observed. Because PCSK6 plays a key role in regulating the activity of NODAL during the development of LR asymmetry in vertebrates [40], we hypothesize that other genes in this pathway are relevant for the development of handedness. Performing gene-set enrichment analysis, we identify an over-representation of SNPs strongly associated with relative hand skill in the human orthologs of genes that cause asymmetry defects when disrupted in mice. We replicate these results in a large independent general population cohort without reading disability. Our results suggest that handedness is a complex trait controlled in part by genes responsible for the establishment of LR body asymmetry early in development.

## Results

The study participants are derived from four cohorts (**Table 1**), three with RD (cohorts 1–3, *n* = 185, 374, and 169 respectively), and a general population cohort (cohort 4, *n* = 2,666). The samples from cohorts 1 and 2 were selected as part of an ongoing study of dyslexia [43], [44]. The samples from cohorts 3 and 4 are part of the Avon Longitudinal Study of Parents and Children (ALSPAC). We divided them into individuals with RD (cohort 3), and those unaffected with RD (cohort 4), based on their IQ and reading scores (see **Materials and Methods**). All have had their relative hand skill assessed.

**Table 1:** GWAS study design.

*Cohort*	*n*	*Genotyping Array*	*Imputation Reference Panel*	*Imputation Software*	*Reading Disabled*
1	185	Illumina 550K	1000 Genomes March 2012	Impute	Yes
2	374	Illumina OmniExpress	1000 Genomes March 2012	Impute	Yes
3	169	Illumina 550K	HapMap Phase II	MACH	Yes
4	2,666	Illumina 550K	HapMap Phase II	MACH	No

Three cohorts with reading disability and a further general population cohort were genotyped and tested after imputation for association with relative hand skill.

### Relative Hand Skill Phenotype

The relative hand skill peg-board task measures the time taken by the subjects to move a row of ten pegs from one location to another with the left hand (L) and right hand (R) separately [26]. Each hand was tested five times, from the average time for each hand we derived the measure PegQ [2(L−R)/(L+R)]. This task produces an approximately normally distributed variable with a positive mean; a positive PegQ indicates superior relative right-hand skill, and a negative PegQ indicates superior relative left-hand skill. In cohort 1, the PegQ distribution has a mean of 0.080 (SD = 0.099) [37], and in cohort 2 it is 0.061 (SD = 0.091). A similar peg-board test involving moving 12 pegs was performed just once (after practice) in ALSPAC (cohorts 3 and 4, see **Materials and Methods**). Since the test was slightly different, the mean PegQ was slightly different to cohorts 1 and 2 (**Table 2**), while the distribution remains unimodal, continuous and approximately normal.

**Table 2:** Individuals with reading disability (cohort 3, *n* = 169) are no more skilled with their left- or right- hand (PegQ) compared to the general population (cohort 4, *n* = 2,666), however they perform the relative hand skill task slower (motor skill).

*Phenotype*	*General Population Ave*	*General Population SD*	*Reading Disabled Ave*	*Reading Disabled SD*	*P*
PegQ	0.11	0.19	0.10	0.18	0.32
Motor Skill	23.57	3.18	24.39	3.65	0.0011

We used a one-tailed Student's t-test, not assuming equal variance.

We investigated the correlation between binary hand preference and relative hand skill using logistic regression in all the individuals in our cohorts for which we have data. The PegQ phenotype is highly correlated with the preferred hand of the participants. In cohorts 1 & 2, for every 1 standard deviation shift to the right of the Peg distribution, individuals are 13.4 times more likely to be right handed (95% Confidence intervals: 6.11–36.49). In ALSPAC (cohorts 3 & 4), they are 3.23 times more likely to be right handed (95% Confidence intervals: 2.83–3.70). The weaker correlation in ALSPAC is likely due to the fact that the peg-board task was performed only once, as opposed to five times in cohorts 1 and 2 (see **Materials and Methods**). As the peg-board tests fine motor control, PegQ also correlates with dexterity, measured as the time taken to perform the task with either the right hand (cohorts 1 & 2, Pearson's r = −0.42, cohorts 3 and 4 r = −0.50) or the left hand (cohorts 1 & 2, Pearson's r = 0.27, cohorts 3 and 4 r = 0.59).

Individuals with RD (cohort 3) are not significantly more likely to be left-handed (*f* = 11.4%) than those in the general population (cohort 4, *f* = 10.9%, two-tailed Fisher's exact test *P* = 0.82). Furthermore, they are not more likely to be shifted toward the left-handed distribution of the peg board task, however their average time for both hands is slower, indicating lower overall motor skill (*P* = 0.001, one tailed Student's t-test, **Table 2**).

### GWAS for Relative Hand Skill

Cohorts 1 and 2 have been genotyped on the Illumina 550K and OmniExpress SNP arrays, respectively, and we imputed genotypes using the 1000 Genomes reference panel (Phase 1, March 2012 release). The samples from cohorts 3 and 4 come from the Avon Longitudinal Study of Parents and Children (ALSPAC) cohort. The participants have performed a similar peg-board task (see **Materials and Methods**), been characterized by similar reading measures to cohorts 1 and 2, been genotyped on the Illumina 550K SNP array, and had genotypes imputed using the HapMap II CEU reference panel. We selected individuals (cohort 3) to closely match the ascertainment criteria for RD used in the other two cohorts. Furthermore we selected a general population cohort, excluding individuals showing phenotypes compatible with a neurodevelopmental disorder diagnosis (cohort 4, consistent with the methodology in our previous report [37]).

**Figure 1:**
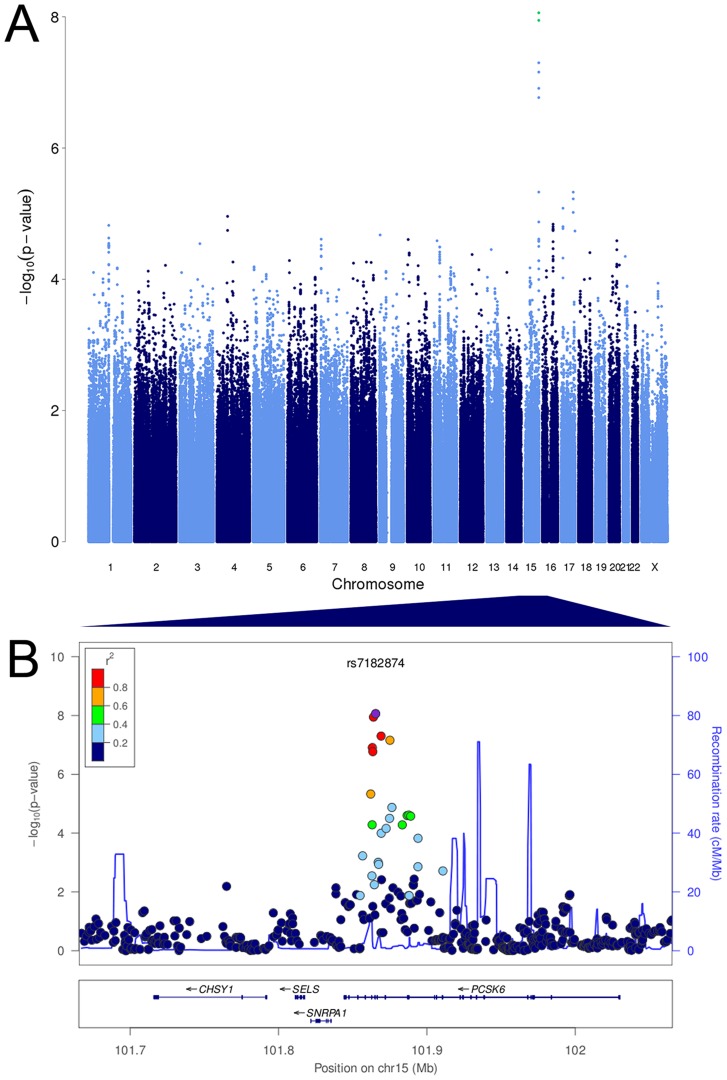
*PCSK6* is associated with relative hand skill in individuals with reading disability. Manhattan plot and visualization of P-values at the *PCSK6* locus. (A) Manhattan plot of the P values for the meta-analysis of the RD Peg relative hand skill GWAS data-sets (cohorts 1–3). The observed P values are plotted against chromosome and position along the genome. The green dots represent markers that are genome-wide significant at P≤5×10^−8^. (B) Negative log_10_ of the P-values for all genotyped and imputed SNPs around *PCSK6* plotted in LocusZoom. Linkage disequilibrium (r^2^) with the most highly associated SNP, rs7182874 (purple diamond), is shown by the color of the SNPs. The recombination rate is shown by the blue line and the locations of genes in this locus are shown in the panel below the plot.

We tested 2,095,209 SNPs for association with relative hand skill in a meta-analysis of the three RD cohorts. The marker rs7182874 within *PCSK6* is the most highly associated SNP (*P* = 8.68×10^−9^, **Figures 1** **, S1,** **Tables 3** **, S1**), confirming our previous findings [37]). Each copy of the minor (derived) allele shifts an individual 0.37 standard deviations toward the right-handed end of the distribution of relative hand skill. No SNP is genome-wide significant in the general population cohort; the most highly associated SNP, rs7883190 (*P* = 2.08×10^−6^) is located approximately 6 Kb upstream of the gene *GPC3* on the X chromosome, a gene that causes heart and lung asymmetry defects when disrupted in mice (cohort 4, **Table S2**) [45].

**Table 3:** The intronic marker rs7182874 (ancestral allele = C; derived, minor allele = T) within the gene *PCSK6* on chromosome 15 is associated with relative hand skill in individuals with reading disability.

*Cohort*	*n*	*P*	*β*	*S.E.*
1	185	2.30E-06	0.55	0.12
2	374	0.0099	0.26	0.10
3	169	0.0049	0.34	0.12
Meta-Analysis	728	8.68E-09	0.37	0.064
General Population (4)	2,666	0.77	−0.01	0.032

β = effect size of each copy of the minor allele in standard deviations, S.E. = Standard Error.

### Gene-Set Enrichment Analysis (GSEA)

Disruption of *PCSK6* in mice causes asymmetry defects [40], [46]. We therefore employed gene-set enrichment analysis (GSEA) to test for an over-representation of highly associated SNPs within the human orthologs of mouse genes whose disruption causes LR asymmetry defects, as annotated by the Mouse Genome Informatics (MGI) resource [47]. GSEA was implemented in MAGENTA, which combines variant association *P* values into individual gene scores, correcting for confounders such as gene size, variant number, and linkage disequilibrium [48]. It then ranks all gene scores and tests for an overrepresentation of a given set of genes above the 75^th^ percentile of all gene *P* values. We chose this cutoff because it yields the best power for detecting enrichment in traits that are highly polygenic with many associations of weak effect [48]. Genes were defined as from 20 Kb upstream of the most 5′ transcription start site to 10 Kb downstream of the most 3′ untranslated region, to include adjacent cis-regulatory elements. We tested for an over-representation of genes annotated to each of 15 morphological asymmetry phenotypes (with between 10 and 100 annotated genes per phenotype) in the meta-analysis of cohorts 1–3. Four phenotype gene sets out of the 15 tested were significant at an FDR≤5% in the combined RD meta-analysis (**Table 4**); abnormal right lung morphology (MP:0009570), double outlet right ventricle (MP:0000284), heterotaxia (MP:0004133), and situs inversus (MP:0002766). MAGENTA also provides the overrepresentation of genes above the 95^th^ percentile cutoff by default. Despite the fact that the expected number of genes above this threshold was very low (typically around one or two genes per phenotype), reducing our power to detect any enrichment, we observed five phenotypes at FDR≤5% (**Table S3**). The genes that comprise the LR asymmetry phenotype sets overlap considerably; thus FDR correction for multiple testing is conservative. A list of the ten most highly associated genes from the four phenotypes above the 75^th^ percentile cutoff is given in **Table 5**. After removing *PCSK6* from the analysis, the phenotypes abnormal right lung morphology, situs inversus, and double outlet right ventricle, still show an over-representation of associated SNPs (FDR≤5%, **Table S4**). Therefore the association in *PCSK6* is not solely driving the results.

**Table 4:** In the relative hand skill GWAS meta-analysis of individuals with reading disability, there is an over-representation of highly associated SNPs within human orthologs of genes involved in causing LR asymmetry phenotypes when knocked out in mice.

*Mouse Phenotype ID*	*Phenotype*	*Genes*	*Median Size/KB*	*Mean Size*	*Expected*	*Observed*	*Adjusted P*
MP:0010429	abnormal heart left ventricle outflow tract morphology	47	42	83	12	13	0.4
MP:0003922	abnormal heart right atrium morphology	20	50	87	5	4	0.74
MP:0009569	abnormal left lung morphology	22	51	89	6	8	0.2
MP:0001706	abnormal left-right axis patterning	62	48	73	16	20	0.17
MP:0009570	abnormal right lung morphology	32	68	120	8	16	0.0087
MP:0000644	Dextrocardia	26	64	113	7	11	0.07
MP:0000284	double outlet right ventricle	87	47	92	22	35	0.016
MP:0002625	heart left ventricle hypertrophy	48	29	107	12	12	0.6
MP:0000276	heart right ventricle hypertrophy	25	18	96	6	3	0.93
MP:0004133	Heterotaxia	42	46	80	11	18	0.025
MP:0000542	left-sided isomerism	17	64	92	4	8	0.068
MP:0004158	right aortic arch	36	27	80	9	14	0.07
MP:0000531	right pulmonary isomerism	15	39	77	4	5	0.35
MP:0000508	right-sided isomerism	16	46	88	4	5	0.37
MP:0002766	situs inversus	24	63	103	6	11	0.046

15 phenotypes that contain between 10 and 100 genes, and cause asymmetry defects when knocked out in mice, were tested using MAGENTA (see **Materials and Methods**). The observed number of genes were compared to the expected using a cut-off that equals the 75^th^ percentile of all gene *P* values. *P* values were then adjusted for multiple testing using an FDR correction.

**Table 5:** LR asymmetry genes are associated with relative hand skill (meta-analysis of cohorts 1–3).

*Gene*	*Chromosome*	*Gene Size/kb*	*Gene P*	*Best SNP*	*SNP P*	*MAF*	*β*
*PCSK6*	15	186	3.94E-08	rs7182874	8.68E-09	0.29	0.37
*PKD2*	4	70	0.00034	rs2728105	0.00015	0.46	0.22
*MNS1*	15	36	0.00087	rs6493858	0.00032	0.44	−0.21
*GLI3*	7	276	0.0056	rs2282920	0.00016	0.41	0.23
*RFX3*	9	301	0.0079	rs2991306	0.00032	0.11	−0.33
*ACVR2B*	3	39	0.017	rs7645704	0.0038	0.07	0.34
*MGRN1*	16	66	0.019	rs2279408	0.0036	0.20	0.20
*LAMA5*	20	58	0.019	rs477859	0.0032	0.22	0.20
*RARA*	17	48	0.025	rs2715553	0.0085	0.47	−0.15
*FZD1*	7	4	0.027	rs6977978	0.0055	0.14	−0.20

We've listed the ten lowest gene *P* values that are also within one of the four enriched phenotypes from **Table 4** in the RD meta-analysis. MAF = minor allele frequency, β = effect size of each copy of the minor allele, in standard deviations.

We took forward the four phenotypes that show an over-representation of genes with low *P*-value SNPs in the RD cohorts for testing in the general population (cohort 4). Three out of the four phenotypes show enrichment at (FDR≤5%), double outlet right ventricle (*P* = 0.019, adjusted for multiple testing), heterotaxia (adjusted *P* = 0.026), and situs inversus (adjusted *P* = 0.021) (**Table S5**). We have therefore shown that our findings from the RD cohort hold true for the general population.

There are 116 unique genes whose disruption in their mouse ortholog is annotated with these three phenotypes. 47 genes in the RD cohorts, and 42 in the general population cohort, show *P* values above the 75^th^ percentile of all gene *P* values. Of these, 16 overlap between the RD meta-analysis and the general population cohort (one-tailed Fisher's exact test, *P* = 0.72). Therefore, although the same phenotypes show enrichments, the associated SNPs and genes are different, i.e. there is both allelic and locus heterogeneity between the cohorts.

Four out of the five most highly associated genes from the mouse phenotypes tested (**Table 5**) are involved in ciliogenesis (*RFX3*, *MNS1*, *GLI3*, and *PKD2*). Because ciliopathies cause cerebral phenotypes like agenesis of the corpus callosum and hydroencephaly, we also tested these two phenotypes in both the meta-analysis of the individuals with RD (cohorts 1–3), and separately in the general population (cohort 4). The absent corpus callosum (MP:0002196) phenotype showed an over-representation in the RD cohorts (adjusted *P* = 0.016, **Table S6**), but not in the general population (cohort 4). Of the associated genes from the absent corpus callosum phenotype, only *GLI3* overlaps with the associated genes in **Table 5**. Hydroencephaly (MP:0001891) did not show an over-representation in either cohort.

## Discussion

We have performed a genome-wide association study (GWAS) meta-analysis for a quantitative measure of handedness and found association with variants that implicate genes involved in the determination of left/right body asymmetry. To achieve this, we developed a novel approach to GWAS pathway analysis, coupling gene-set enrichment analysis (GSEA) with mouse phenotype data. In the RD meta-analysis, we showed there was an over-representation of variants with low *P*-values within human orthologs of genes that cause LR asymmetry phenotypes when knocked-out in mice. We replicated the same over-representation in the general population. We also confirmed that the gene *PCSK6* was associated with relative hand skill at genome-wide significance (RD meta-analysis, cohorts 1–3, rs7182874 *P* = 8.68×10^−9^).

The genes identified by GSEA in the meta-analysis of cohorts with RD are involved in the determination of LR asymmetry in mice (**Table 5**). *Mns1* and the transcription factor *Rfx3* both play key roles in ciliogenesis [46], [49]. The transcription factor *Gli3* is expressed at primary cilia, localizing to both the distal tip of the cilia as well as the nucleus, and is involved in Sonic hedgehog signal transduction [50]. The unidirectional fluid flow created by cilia rotation that breaks asymmetry is detected via the Ca^2+^ channel PKD2 [51]. This flow concentrates the morphogen NODAL on the left, which is cleaved into an active form by the protease PCSK6 [52]. The NODAL signal is then transduced through the transmembrane receptor ACVR2B [53]. Retinoic acid acts through receptors like RARA to affect expression of *Nodal*, *Pitx2*, and *Lefty* in mice [54]. FZD1 also acts upstream of NODAL, it is a receptor for WNT3A [55], which activates the Delta/Notch pathway to regulate expression of *Nodal* in mice [56]. Similarly, in a significant proportion of mouse embryos where *Mgrn1* is disrupted, *Pitx2*, *Lefty1*, and *Lefty2* are abnormally expressed [57]. LAMA5 is a component of the extracellular matrix, its precise role in the development of LR asymmetry is unknown. However, mice in which *Lama5* has been disrupted develop Polycystic Kidney Disease (PKD) [58], a phenotype comorbid with LR asymmetry defects caused by ciliopathies [59].

We have shown that our findings from the RD cohorts hold true for the general population, three of the four phenotype gene sets tested are significant at FDR≤5% (heterotaxia, situs inversus, and double outlet right ventricle). However, the associated variants and genes are different. For example, the most highly associated marker in the general population GWAS (rs7883190, *P* = 2.08×10^−6^, **Table S2**) is located 6 Kb upstream of *GPC3* on the X chromosome, a gene that causes the double outlet right ventricle phenotype when disrupted in mice [45].

If genes that are critical to the breaking of LR body symmetry influence handedness and brain asymmetry, individuals with Mendelian disorders that cause asymmetry defects like situs inversus (SI) should have a greater prevalence of left-handedness. However, studies dating as far back as 1836 have shown that individuals with SI present with a prevalence of left-handedness equal to the rest of the population [60]–[62]. A more recent study of individuals with primary ciliary dyskinesia (a ciliopathy that causes SI) has confirmed this finding [63]. However, two brain imaging studies of individuals with SI have shown a significant reversal of the typical pattern of right-frontal and left-occipital petalia asymmetry [64], [65], of which one study also showed a significant reversal of language dominance [65]. However, each study only assessed three individuals with SI, and given its rarity (∼1 in 10,000 [62]), large-scale studies have yet to be performed. Furthermore, a study of gene expression in fetal brains identified 27 differentially expressed genes between the two hemispheres, none of which are known to be involved in body asymmetry development [66]. It is possible that compensatory mechanisms allow for the normal development of handedness in individuals with situs inversus and the development of handedness and brain asymmetry is more complex than just involving early LR asymmetry determining genes.

To help explain the relationship between LR asymmetry determination, brain asymmetry, and handedness, we hypothesize that the same molecular mechanism for visceral LR asymmetry is involved during cerebral development. In support of this, RFX3 regulates the expression of GLI3 in the telencephalon in mice, which in turn regulates the distribution of guidepost neurons necessary for corpus callosum formation [67]. *Rfx3* deficient mice show corpus callosum agenesis and disorganization of guidepost neurons necessary for axon pathfinding across the brain's midline [67]. Humans with ciliopathies commonly present with two specific structural changes within the brain; hydroencephaly and an absent corpus callosum [59]. Because four of the five most strongly associated genes shown in **Table 5** are involved in ciliogenesis, we therefore performed GSEA on these two phenotypes as well. Enrichment was detected in absent corpus callosum, but not in hydroencephaly in the RD cohort, although this did not replicate in the general population cohort. The only gene that overlaps with the LR asymmetry genes in **Table 5**, annotated to the absent corpus callosum phenotype, is *GLI3*. However, *RFX3* has not yet been annotated to this phenotype in the mouse phenotype ontology [67], while *PCSK6* is also known to have high expression in the corpus callosum [68]. Both postmortem measurements and MRI studies have independently indicated the tendency for the corpus callosum to be larger in left-handers [69], [70], suggesting that the corpus callosum plays a role in the expression of handedness. Our study provides genetic evidence in support of this.

The relationship between these genes and both corpus callosum development and brain asymmetry are not well understood in humans. However, infants with agenesis of the corpus callosum appear to show a similar bias in right-handedness and performance on unimanual hand skill tasks to controls, although they perform slower at bimanual tasks [71], [72]. Brain asymmetries have been better studied in zebrafish and have revealed that these processes are under the control of Nodal-related genes, suggesting a conserved pathway controlling both visceral and cerebral asymmetries [73]. A particular line of zebrafish, *frequent situs inversus (fsi)*, displays reversal of both body and neuronal asymmetries, as well as of some characteristic behavioral phenotypes, like eye preference and direction of prey approach [74]. Our study implicates Nodal-related genes in the development of handedness and therefore brain asymmetry in humans.

The main limitations of this study are dependent on the currently available datasets, characterized by a degree of genotype and phenotype heterogeneity, and a small sample size. Genotypes from ALSPAC were imputed with HapMap phase II instead of the 1000 genomes reference panel. This means we were restricted to analyzing only ∼2 million SNPs instead of over 6 million common SNPs in the 1000 genomes project. The imputation accuracy is also lower with HapMap II, however we restricted our study to common variants (MAF≥5%), for which imputation accuracy is high (see box 4 of [75]). Not only was the relative hand skill phenotype slightly different in ALSPAC (see **Materials and Methods**), it was only measured once instead of five times. Furthermore the phenotype is only a proxy for one specific aspect of handedness, namely manipulating objects quickly with each hand separately. It is strongly correlated with both hand preference and dexterity (see **Results**), and it is not clear whether the associations observed are due to handedness or dexterity or a combination of the two. Although we interrogated a quantitative phenotype, which is more powerful than a binary case-control GWAS such as that performed by Eriksson et al. [35], the numbers of participants were relatively small (discovery cohorts: *n* = 744 and replication: *n* = 2,666). Analysis of phenotypes like PegQ in much larger cohorts will be needed to investigate comprehensively the biological complexity of laterality.

Based on our results we propose that the same biological mechanism underlying the development of LR asymmetry in the body plays a role in determining handedness. However, although we replicated the same LR asymmetry phenotypes in the general population as the RD cohorts, different SNPs located in different genes within these same pathways were responsible for driving the association. Furthermore, the genome-wide significant SNP in *PCSK6* does not even show the same trend in the general population (**Table 3**). This suggests there is both allelic and locus heterogeneity between the RD and general population cohorts. We suggest this heterogeneity may derive from epistasis between genes involved in dyslexia and those involved in handedness. In support of this, a cilia-related coexpression module derived from publically available microarray datasets found that the dyslexia associated genes, *DCDC2*, *DYX1C1*, and *KIAA0319* are coexpressed in cilia, suggesting they interact with genes involved in the determination of LR asymmetry [76]. Furthermore, overexpression of *Dcdc2* increases the length of cilia in rats [77]. Also, the Zebrafish ortholog of the dyslexia candidate gene *DYX1C1* has been shown to play a key role in cilia growth and function, and the inhibition of *dyx1c1* produces asymmetry phenotypes like situs inversus [78]. We have confirmed, as previously reported [16], [23], that no consistent association has been found between either hand preference or relative hand skill and dyslexia. However, early work by Galaburda et al. reported eight consecutive post-mortem specimens with RD that had reduced planum temporale asymmetry [79], [80]. This suggests subtle structural asymmetries might be involved in dyslexia. It has also been shown that reading performance is correlated with motor abilities on the peg-board test [23], [81]. We have replicated this finding by comparing cohorts 3 and 4 in the current study – demonstrating that individuals with RD are slower at the peg board test than those in the general population (*P* = 0.001, **Table 2**). This is consistent with a broad literature showing motor skill difficulties in children with dyslexia [82]–[86].

The precise relationship between handedness, cerebral asymmetry, and neurodevelopmental disorders like dyslexia remains to be determined. However, our results have implicated some of the same genes involved in body asymmetry in handedness, and therefore the development of cerebral asymmetry in both individuals with RD and the general population. Larger scale GWAS or sequencing studies of handedness, coupled with a deeper understanding of the genetic basis of language-related disorders like dyslexia will help determine whether handedness and language do indeed develop hand-in-hand.

## Materials and Methods

### Ethics Statement

Ethical approval for cohorts 1 and 2 was acquired from the Oxfordshire Psychiatric Research Ethics Committee (OPREC O01.02). For the ALSPAC cohorts, ethical approval was obtained from the ALSPAC Law and Ethics committee and relevant local ethics committees, and written informed consent provided by all parents.

### Study Participants

The initial cohort was selected for severity of phenotype to undergo a GWAS for RD as described before [37]. Briefly, families were recruited from the Dyslexia clinic of the Royal Berkshire Hospital and one child per family with dyslexia was selected (*n* = 185) ranging in age from 6 to 25 years. Unrelated individuals with RD (cohort 2, *n* = 374) were recruited from either the Dyslexia Research Centre clinics in Oxford and Reading or the Aston Dyslexia and Development Clinic in Birmingham [43]. The majority of these individuals are between 8 and 18 years old. The third cohort of unrelated individuals came from the Avon Longitudinal Study of Parents and Children (ALSPAC). ALSPAC is a general population cohort that started collecting data on around 14,000 pregnant women in the Bristol area with a then expected delivery date between 1 April 1991 and 31 December 1992 [87]. This cohort is described in detail on the website (http://www.alspac.bris.ac.uk) and elsewhere [88]. The children are being followed and measured for many different phenotypes including laterality measures. For the present study, we assigned individuals from the ALSPAC cohort into a sub-group with RD (*n* = 169, cohort 3), and a sub-group representing the general population who do not show phenotypes compatible with a neurodevelopmental disorder (*n* = 2666) consistent with our previous study [37].

### Relative Hand Skill Phenotype

The peg-board test [26] was administered to the individuals who were analyzed in cohorts 1 and 2. The relative hand skill peg-board task measures the time taken by the subjects to move a row of ten pegs from one location to another with the left hand (L) and right hand (R) separately [26]. Each hand was tested five times, from the average time for each hand we derived the measure PegQ [2(L−R)/(L+R)]. In cohort 1, the distribution has a mean of 0.080 (SD = 0.099) [37], and in cohort 2 it is 0.061 (SD = 0.091). A similar peg-board test was performed in ALSPAC (cohorts 3 and 4) as part of a battery of manual dexterity tests known as Movement ABC [89]. In this case there were 12 pegs on the table that the child picked up one-at-a-time and placed in a peg-board. After initial practice, children performed the test once (due to time constraints) with each hand. Since the test was slightly different, the mean PegQ was slightly different to cohorts 1 and 2 (**Table 2**), while the distribution remains unimodal, continuous and approximately normal. One outlier was removed from further analysis (PegQ = 1.50).

### Genotyping and Quality Control

Genotypes for cohort 1 were generated on the Illumina 550K SNP array [37]. Genotypes for cohort 2 were generated for the present study on the Illumina OmniExpress SNP array (730,525 markers), according to the manufacturer's instructions. We followed the quality control protocol set out by Anderson et al., 2010 [90] excluding individuals with: discordant sex information, a low genotyping success rate (≤98%, **Figure S2**), duplicated or related to other individuals (identity by state IBS metric ≥0.1875), divergent ancestry from CEU hapmap3 reference samples based on principal component analysis (PCA, **Figure S3**).

Furthermore, we excluded SNPs that: have an excessive missing genotype rate (call rate ≤0.98, **Figure S4**), show a significant deviation from Hardy-Weinberg Equilibrium (*P*≤10^−6^), and have a minor allele frequency (MAF)≤0.05.

After QC, 458,236 (cohort 1) and 589,768 (cohort 2) single nucleotide polymorphisms (SNPs) were taken forward for imputation.

Genotyping for the ALSPAC (cohorts 3 and 4) was carried out using the Illumina HumanHap 550K array. Individuals were excluded on the basis of the following: sex mismatches, minimal or excessive heterozygosity, disproportionate levels of individual missingness, cryptic relatedness measured as proportion of identity by descent, and insufficient sample replication. The remaining individuals were assessed for evidence of population stratification by multidimensional scaling analysis and compared with Hapmap II (release 22) European descent (CEU), Han Chinese, Japanese and Yoruba reference populations; all individuals with non-European ancestry were removed in the ALSPAC dataset. SNPs with a minor allele frequency of <1%, a call rate of <95% or evidence for violations of Hardy-Weinberg equilibrium were removed.

### Imputation

In cohorts 1 and 2, haplotypes were inferred prior to imputation using the Segmented HAPlotype Estimation and Imputation Tool (shapeit v2 – http://www.shapeit.fr/ [91]). We used IMPUTE [42] (v2.2.2 – http://mathgen.stats.ox.ac.uk/impute/impute_v2.html) to estimate the genotypes of SNPs not directly genotyped. Imputed SNPs were filtered out if they had an MAF≤0.05 or imputation info ≤0.8. We then used the 1000 Genomes Phase I integrated variant set (March 2012, build 37) as the reference panel for imputation, testing 5,829,682 SNPs for association.

In ALSPAC autosomal genotypic data were imputed in ALSPAC using Markov Chain Haplotyping software (MACH v.1.0.16) and phased haplotype data from CEU individuals (Hapmap release 22, Phase II NCBI B36, dbSNP 126) based on a cleaned dataset of 8,365 individuals and 464,311 autosomal SNPs. After imputation, all poorly imputed SNPs (RSQR<0.30) and those with low minor allele frequency (MAF≤0.01) were excluded from analyses.

We were not able to obtain 1000 Genomes imputation data for the ALSPAC cohort, however we did perform imputation using the HapMap II reference panel for cohorts 1 and 2, and obtained very similar results, suggesting our overall conclusions are not an artifact of using different imputation panels (**Tables S7, S8**).

### Statistical Analysis

Association with PegQ was tested under an additive model using SNPTEST (v2.4.1, http://www.stats.ox.ac.uk/~marchini/software/gwas/snptest.html) simultaneously specifying sex and age as covariates. The genomic inflation factor (*λ*) was calculated for directly genotyped and imputed SNPs combined, and showed no evidence of population structure, *λ* was 1.01 for cohort 1, 1.00 for cohort 2, 1.01 for cohort 3, and 1.00 for cohort 4. The values of PegQ were standardized in all cohorts separately to give a mean of 0, and a SD of 1. The results for the three cohorts were then meta-analyzed under a fixed-effects model with inverse variance weighting using GWAMA [92]. In total, 2,095,209 SNPs that were either directly genotyped or imputed and overlapped in all three studies and passed quality control were tested for association.

We performed pathway analysis using Meta-Analysis Gene-set Enrichment of variaNT Associations (MAGENTA) [48]. MAGENTA first maps SNPs to genes (defined as from 20 Kb upstream of the 5′ UTR to 10 Kb downstream), and then selects the most strongly associated SNP, after correcting for confounding factors like gene size, the number of SNPs, linkage disequilibrium, number of recombination hotspots, in order to calculate a *P* value for each gene. It then ranks all gene *P* values and tests if there is an overrepresentation of a given set of genes above a user-specified cutoff of all genes. We chose the 75^th^ percentile as a cutoff because it yields the best power for detecting enrichment in traits that are highly polygenic with many associations with weak effect [48]. We tested all pathways in the mouse genome informatics database (MGI, *n* = 15) that cause asymmetry defects when knocked out in mice and contain more than 10 and less than 100 genes, against a background of all genes in the MGI database (6,573 genes as of August 2012, http://www.informatics.jax.org/ [47]). Mouse phenotypic measurements were chosen because they closely resemble observations from human clinical examination of patients with mutations in orthologous genes [93].

Finally, in order to compare relative hand skill and hand motor skill in cohorts 3 and 4, we used a one-tailed Student's T-Test, not assuming equal variance.

## Supporting Information

Figure S1 QQ plot of the P values for the relative hand skill GWAS meta-analysis in individuals with reading disability (RD). The observed *P* values are plotted against the expected *P* values; a deviation from the null (diagonal line) indicates an enrichment of genuine associations.
(PDF)

Figure S2 Genotype failure rate plotted against heterozygosity. Shading indicates sample density and dashed lines denote quality control thresholds.
(PDF)

Figure S3 Principal component analysis. Ancestry Clustering based on principle component analysis of the original GWAS data. HapMap3 reference samples; CEU (red), JPT & CHB (purple) and YRI (green), are plotted alongside all individuals in the GWAS (black crosses). Individuals with a second principal component score less than 0.072 (black dashed line) were removed from the study.
(PDF)

Figure S4 SNP call rate. Histogram of the fraction of missing data for each SNP across all individuals that passed the ‘per-individual’ quality control (see Materials and Methods). The dashed vertical line represents the threshold (2%) at which SNPs were removed from subsequent analysis because of an excess failure rate.
(PDF)

Table S1 A list of SNPs with *P*≤1×10^−4^ from the relative hand skill GWAS meta-analysis in individuals with RD (*n* = 728). All the genes within 100 kb to the left or right of the SNP are listed, if no gene is present, it is denoted by a dash. EAF = Effect Allele Frequency, β = effect size (standard deviation shift per copy of the effect allele, 95 percent confidence intervals included), SE = standard error.
(XLSX)

Table S2 A list of SNPs with *P*≤1×10^−4^ in the relative hand skill GWAS in the general population (n = 2,666). All the genes within 100 kb to the left or right of the SNP are listed, if no gene is present, it is denoted by a dash. EAF = Effect Allele Frequency, β = effect size (standard deviation shift per copy of the effect allele, 95 percent confidence intervals included), SE = standard error.
(XLSX)

Table S3 Fifteen phenotypes that contain between 10 and 100 genes, and cause asymmetry defects when knocked out in mice, were tested using MAGENTA for an enrichment of SNPs with low *P* values. The observed number of genes were compared to the expected using a cut-off that equals the 95^th^ percentile of all gene *P* values (compare to the 75^th^ percentile cutoff in Table 4). *P* values were then adjusted for multiple testing using an FDR correction.
(XLSX)

Table S4 After removing the known association in *PCSK6* from the analysis, in the relative hand skill GWAS meta-analysis of individuals with reading disability, there remains an over-representation of highly associated SNPs within human orthologs of genes involved in causing LR asymmetry phenotypes when knocked out in mice. Fifteen phenotypes that contain between 10 and 100 genes, and cause asymmetry defects when knocked out in mice, were tested for an enrichment of SNPs with low *P* values. We used an enrichment cut-off that equals the 75^th^ percentile of all gene *P* values. *P* values were then adjusted for multiple testing using an FDR correction.
(XLSX)

Table S5 In the relative hand skill general population GWAS, there is an over-representation of highly associated SNPs within human orthologs of genes involved in causing LR asymmetry phenotypes when knocked out in mice. We tested four phenotypes that contain between 10 and 100 genes, causing asymmetry defects when knocked out in mice, and showed enrichment of SNPs with low *P* values in the RD cohorts meta-analysis. We used an enrichment cut-off that equals the 75^th^ percentile of all gene P values. P values were then adjusted for multiple testing using an FDR correction.
(XLSX)

Table S6 In the relative hand skill GWAS meta-analysis of individuals with reading disability, there is an over-representation of highly associated SNPs within human orthologs of genes involved in causing an absent corpus callosum when knocked out in mice. We tested two cerebral phenotypes that are often observed in individuals with ciliopathies for an enrichment of SNPs with low *P* values. We used an enrichment cut-off that equals the 75^th^ percentile of all gene *P* values. *P* values were then adjusted for multiple testing using an FDR correction.
(XLSX)

Table S7 GWAS results based on imputations from HapMap data. A list of SNPs with *P*≤1×10^−4^ from the relative hand skill meta-analysis in individuals with RD (*n* = 728). All the genes within 100 kb to the left or right of the SNP are listed, if no gene is present, it is denoted by a dash. EAF = Effect Allele Frequency, β = effect size (standard deviation shift per copy of the effect allele, 95 percent confidence intervals included), SE = standard error. Results are very similar to those obtained using 1000 Genomes data for imputation (see Table S1).
(XLSX)

Table S8 MAGENTA pathway analysis based on imputations from HapMap data. Fifteen phenotypes that contain between 10 and 100 genes, and cause asymmetry defects when knocked out in mice, were tested using MAGENTA for an enrichment of SNPs with low *P* values. *P* values were then adjusted for multiple testing using an FDR correction. Results are very similar to those obtained using 1000 Genomes data for imputations (see Table 4).
(XLSX)
