# Supplementary figures and images for: Common Variants in Left/Right Asymmetry Genes and Pathways Are Associated with Relative Hand Skill
Source: PLoS Genet. 2013 Sep 12;9(9):e1003751. doi: 10.1371/journal.pgen.1003751 (PMC3772043; doi:10.1371/journal.pgen.1003751)

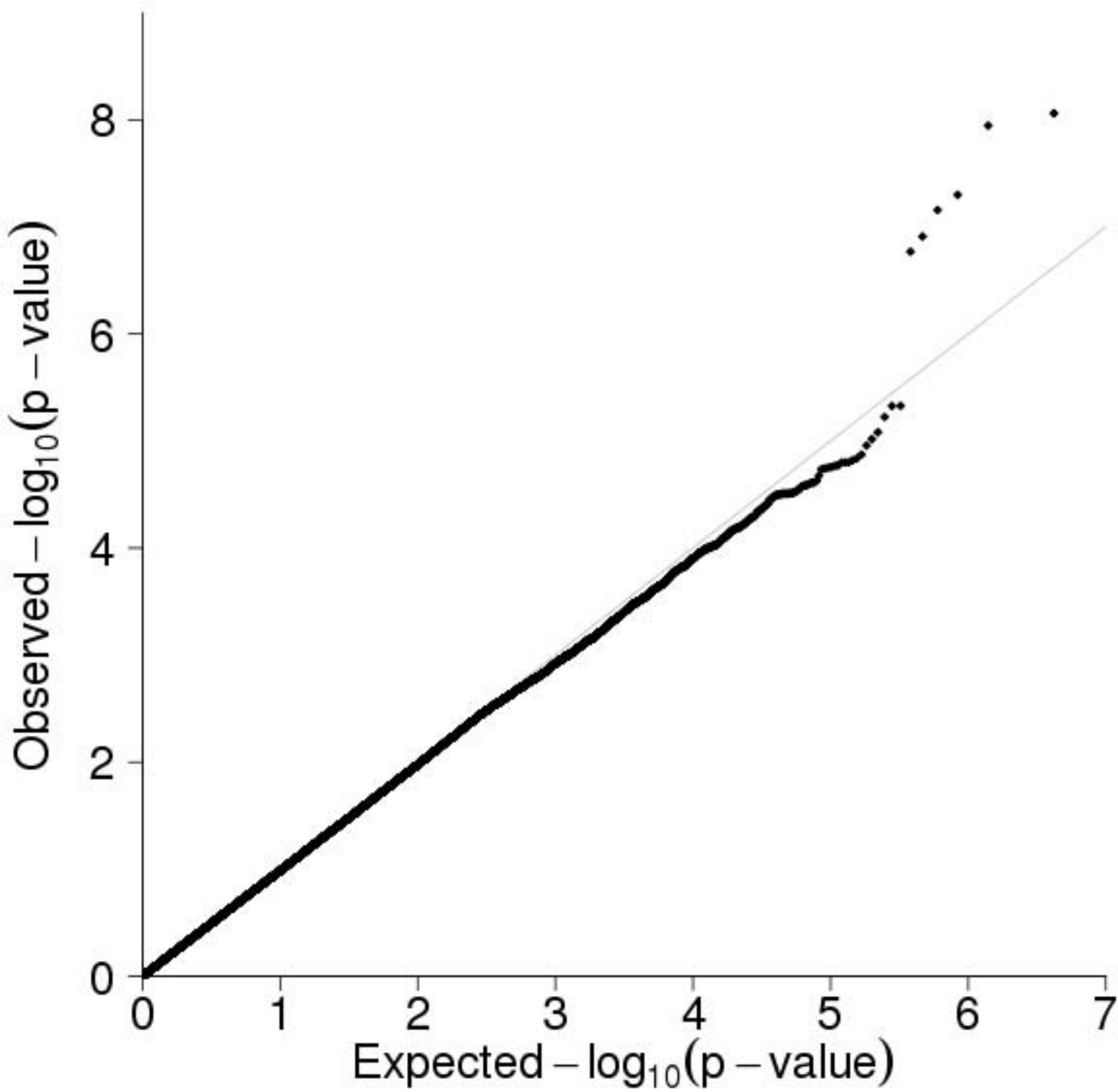

Supplement: Figure S1 — QQ plot of the P values for the relative hand skill GWAS meta-analysis in individuals with reading disability (RD). The observed P values are plotted against the expected P values; a deviation from the null (diagonal line) indicates an enrichment of genuine associations. (PDF) [file pgen.1003751.s001.pdf]

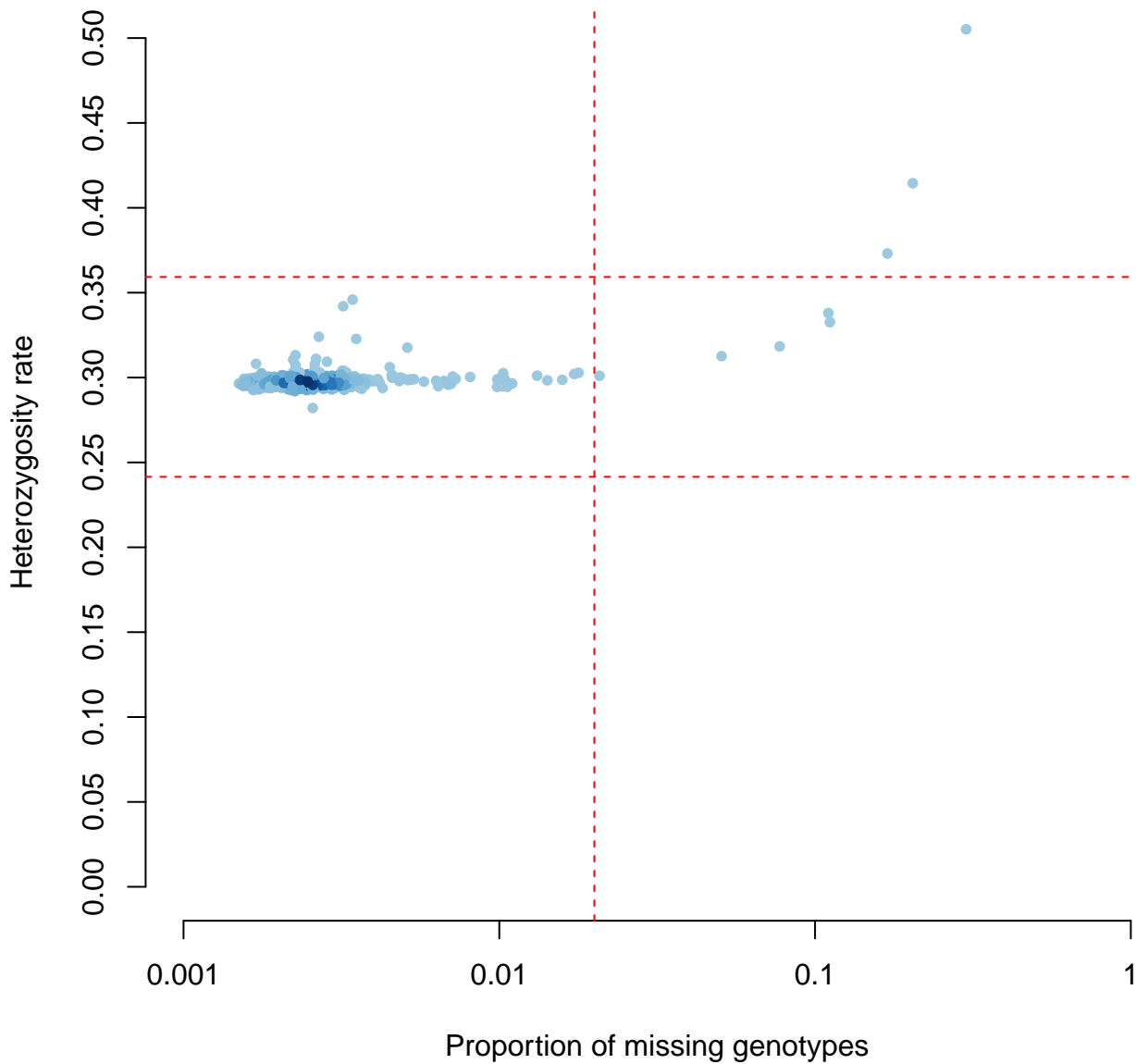

Supplement: Figure S2 — Genotype failure rate plotted against heterozygosity. Shading indicates sample density and dashed lines denote quality control thresholds. (PDF) [file pgen.1003751.s002.pdf]

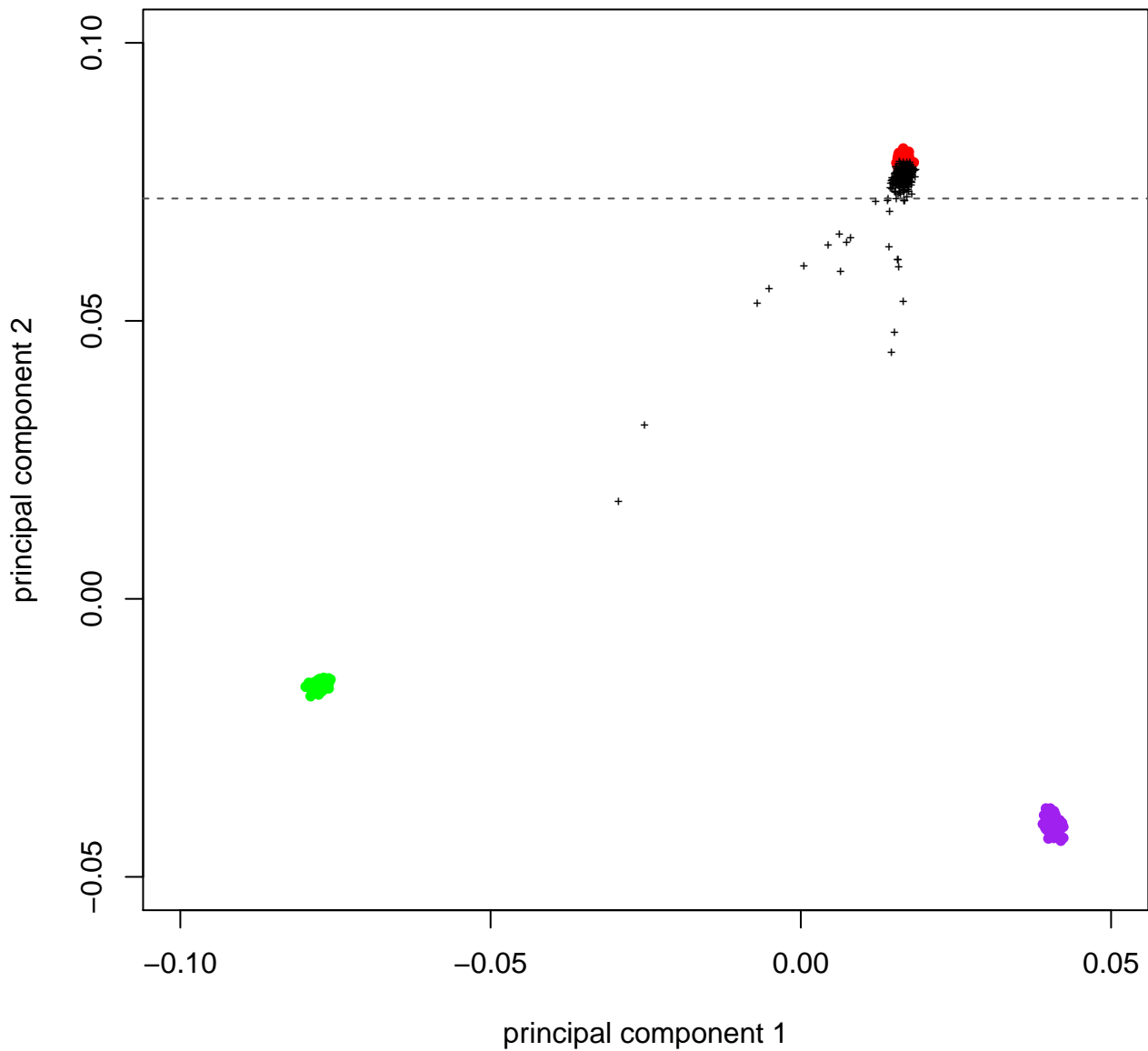

Supplement: Figure S3 — Principal component analysis. Ancestry Clustering based on principle component analysis of the original GWAS data. HapMap3 reference samples; CEU (red), JPT & CHB (purple) and YRI (green), are plotted alongside all individuals in the GWAS (black crosses). Individuals with a second principal component score less than 0.072 (black dashed line) were removed from the study. (PDF) [file pgen.1003751.s003.pdf]

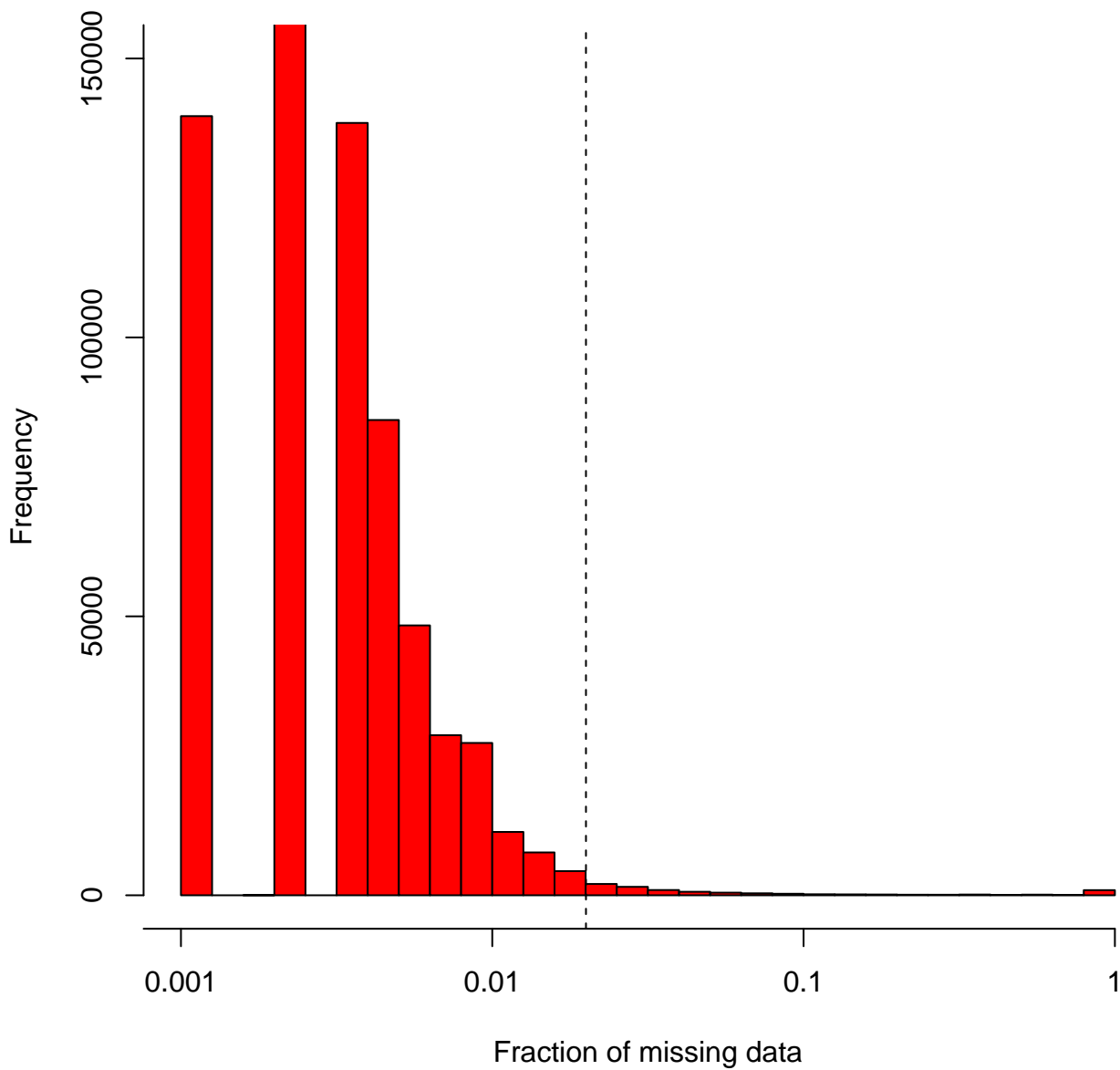

Supplement: Figure S4 — SNP call rate. Histogram of the fraction of missing data for each SNP across all individuals that passed the ‘per-individual’ quality control (see Materials and Methods). The dashed vertical line represents the threshold (2%) at which SNPs were removed from subsequent analysis because of an excess failure rate. (PDF) [file pgen.1003751.s004.pdf]
